# Drug Utilization on Neonatal Wards: A Systematic Review of Observational Studies

**DOI:** 10.3389/fphar.2017.00027

**Published:** 2017-02-08

**Authors:** Rosliana Rosli, Ahmad Fauzi Dali, Noorizan Abd Aziz, Amir Heberd Abdullah, Long Chiau Ming, Mohamed Mansor Manan

**Affiliations:** ^1^Department of Pharmacy Practice, Faculty of Pharmacy, Universiti Teknologi MARASelangor, Malaysia; ^2^Department of Environmental Health, Faculty of Health Sciences, Universiti Teknologi MARABertam, Malaysia; ^3^Unit for Medication Outcomes Research and Education, Pharmacy, School of Medicine, University of TasmaniaHobart, Australia; ^4^School of Pharmacy, KPJ Healthcare University CollegeNilai, Negeri Sembilan, Malaysia

**Keywords:** health services administration, drug utilization review, hospital central supply, daily defined dosage, drug dosage calculations

## Abstract

Despite limited evidence on safety and efficacy of drug use in neonates, drugs are extensively used in this age group. However, the availability of information on drug consumption in neonates, especially inpatient neonates, is limited. This paper systematically reviews published studies on drug utilization in hospitalized neonates. A systematic literature review was carried out to identify observational studies published from inception of databases used till August 2016. Four search engines, namely Medline, CINAHL, Embase, and PubMed, were used. Publications written in English that described drug utilization in neonatal wards were selected. Assessment of the data was based on the category of the study design, the objective of study and the method used in reporting drug consumption. A total of 20 drug utilization studies were identified, 12 of which focused on all drug classes, while the other eight evaluated antimicrobials. Studies were reported in Europe (*n* = 7), the United States (*n* = 6), India (*n* = 5), Brazil (*n* = 1), and Iran (*n* = 1). Substantial variance with regard to study types (study design and methods), data source, and sample size were found among the selected studies. Of the studies included, 45% were cross-sectional or retrospective, 40% were prospective studies, and the remaining 15% were point prevalence surveys. More than 70% of the studies were descriptive studies, describing drug consumption patterns. Fifteen per cent of the descriptive studies evaluated changes in drug utilization patterns in neonates. Volume of units was the most prevalent method used for reporting all drug categories. The ATC/DDD system for reporting drug use was only seen in studies evaluating antimicrobials. The most commonly reported drugs across all studies are anti-infectives for systemic use, followed by drugs for the cardiovascular system, the nervous system and the respiratory system. Ampicillin and gentamicin were the most prescribed antimicrobials in hospitalized neonates. The present review reveals that neonates are exposed to a high number of drugs and various methods are used to report drug consumption in this age group. The best measure of drug consumption to quantify prevalence of drug use in neonates remains to be identified and additional research in this area is warranted.

## Introduction

The World Health Organization (WHO) defined Drug Utilization Research (DUR) as research into the marketing, distribution, prescription, and use of drugs in society, with special emphasis on the resulting medical, social, and economic consequences (World Health Organization, 2003). Besides describing patterns of drug consumption, DUR can also be used to identify problems related to drugs that deserve more research in various health care settings. Drug utilization monitoring can be used to assess the rational use of drug therapy when data on drug use is correlated with figures on morbidity, outcome of treatment (effectiveness in clinical and economic terms) and quality of care (Zuppa et al., 2007; Wettermark et al., 2016).

As monitoring of drug consumption can identify problems related to drug therapy, evidence from DUR is essential to create awareness about irrational drug use by giving feedback to physicians and recommending measures to improve prescribing behavior (Chatterjee et al., 2007; Sequi et al., 2013). Rational and appropriate prescribing is especially crucial for neonates due to their non-fully developed organ functions and the small body size that predisposes them to drug toxicity if an overdose occurs. Most of the drugs in the market were not tested for use in this age group as neonates were often excluded during clinical trials. Therefore, there is limited information on the safety and efficacy of drugs used to treat this population (Smyth and Weindling, 1999; Chatterjee et al., 2007; Sammons, 2011; Nor Aripin et al., 2012; Sequi et al., 2013).

Neonates, particularly from high-risk pregnancies, often present with multiple co-morbidities due to immature organs, which may necessitate intensive and complex medical care with high exposure to drugs. Due to limited clinical trial data among neonates, pediatricians extrapolate suitable drug dosing from adult patients leading to unlicensed or off-label use of drugs among neonates (Sanghera et al., 2006; Awaisu and Sulaiman, 2007; Sturkenboom et al., 2008; Dessi et al., 2010; Neubert et al., 2010; Lass et al., 2011; Booth et al., 2012; Kimland and Odlind, 2012; Ballard et al., 2013). Advances in neonatology have not only improved the survival of neonates but have contributed to dynamic changes in the drug utilization profile in neonates, both in the number of drugs and the pharmacotherapeutic groups (Clark et al., 2006; Warrier et al., 2006; Chatterjee et al., 2007; Hsieh et al., 2014). Despite the advances in this area, there is a paucity of information on drug utilization in neonatals, particularly in the hospital setting (Du et al., 2006; Neubert et al., 2010).

To the best of our knowledge, only a handful of systematic reviews have been conducted to evaluate drug utilization in the pediatric population, and these are mainly focused on outpatient settings. A review by Clavenna and Bonati evaluated 128 studies of pediatric drug utilization in outpatient settings published between January 1994 and December 2008, whilst others reviewed drug utilization of specific drug classes (e.g., antibiotics, anti-asthmatics, and antidepressants, Rossignoli et al., 2007; Clavenna and Bonati, 2009; Bianchi et al., 2010). Other reviews reported statistical methods performed in DUR using the same studies included in the previous review by Clavenna and Bonati (2009) and updated the literature search up to December 2011 (Clavenna and Bonati, 2009; Sequi et al., 2013). A global extant literature search confirms a gap in current medical knowledge. Thus, the aim of this review is to determine the drug prescribing patterns for neonates in hospital, with a focus on the most common therapeutic class prescribed, and to evaluate different methodologies used in reporting drug consumption in hospitalized neonates. A search of the literature was therefore performed to explore the drug utilization profile for inpatient neonates.

## Methods

### Search strategy

**Figure 1:**
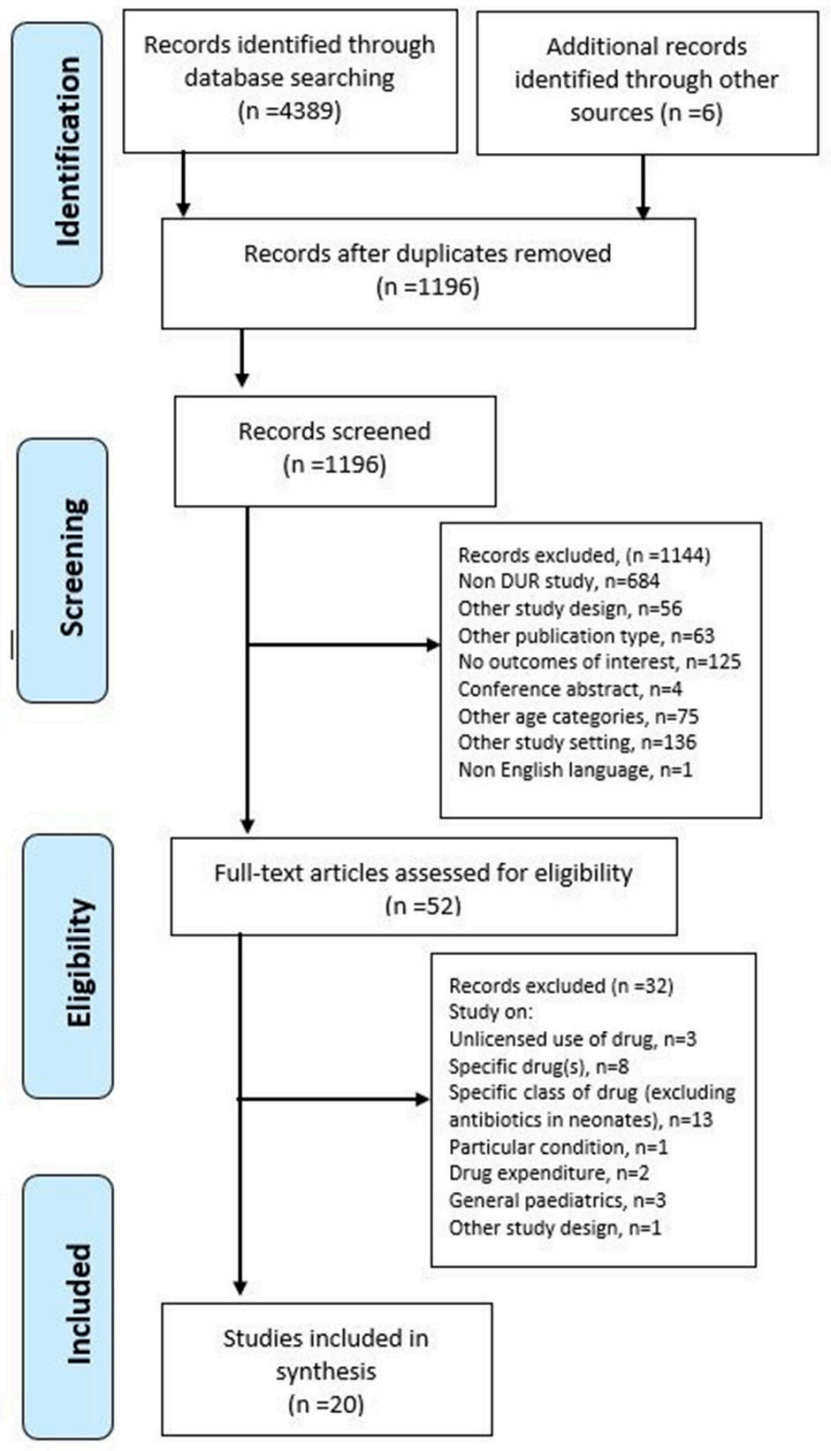
**Flow chart of the systematic review**.

A systematic search was performed in August 2016 on Medline (1946–2016), CINAHL (1937–2016), Embase (1947–2016), and PubMed (1996–2016) for all articles written in English reporting on drug utilization in neonatal wards from inception of the database to August 2016. The search was restricted to human studies and those that described drug utilization or drug patterns or trends. The Preferred Reporting Items for Systematic Reviews and Meta-Analyses (PRISMA) flowchart (Figure 1) and a PRISMA checklist (Refer to S1 PRISMA Checklist) are used to illustrate the electronic search progression of this study (Moher et al., 2009).

### Search terms

The search was performed using the Boolean operators (“AND” & “OR”) with a combination of neonate(s) or newborn, infants with the following key words: drug utilization, defined daily dose(s), prescribed daily dose(s), and anatomical therapeutic chemical classification (ATC). The detailed search strategies used to retrieve articles from the databases are shown in Table 1.

**Table 1:** **Search strategy used to search publications from databases**.

**Keywords**	**Search engines**	**Results**	**Chosen**
(“drug utilization” OR “drug utilisation” OR “defined daily dose^*^” OR “prescribed daily dose^*^” OR “anatomical therapeutic chemical classification”) AND (“infant^*^” OR “neonate^*^” OR “newborn”)	Ovid (Medline)	1060	42
drug utilization OR drug utilisation OR defined daily dose OR prescribed daily dose OR anatomical therapeutic chemical classification AND infant OR neonate OR newborn	EBSCHO host (CINAHL Plus)	365	0
drug NEXT/2 utilisation OR “prescribed daily dose^*^” OR “defined daily dose^*^” OR “anatomical therapeutic chemical classification” AND “neonate^*^” OR “newborn”/exp OR “newborn” OR “infant”/exp OR ‘infant”	Embase	621	4
(drug utilization OR drug utilisation OR defined daily dose OR prescribed daily dose OR anatomical therapeutic chemical classification) AND (infant OR neonate OR newborn)	PubMed	2289	0

### Inclusion criteria

Studies were included if they reported any measures of drug consumption: drug volume or expenditure, Defined Daily Doses (DDD), Prescribed Daily Doses (PDD), and categories of drugs (Anatomical Therapeutic Chemical Classification (ATC). The age categories for studies to be selected was defined on the basis of the International Conference on Harmonisation Guidelines on the Clinical Investigation of Medicinal Products in the Paediatric Population: neonates ≤ 27 days and infants ≤ 23 months (International Conference on Harmonisation of Technical Requirements for Registration of Pharmaceuticals for Human Use, 2014). Another inclusion criterion is infants in the neonatal wards, as there were critically ill neonates who required medical support, which consequently led to longer stays in the neonatal ward, meaning that the children surpassed their neonatal age. Studies on drug utilization in the general hospitalized pediatric population were only included if neonate drug consumptions were reported separately from other age groups and the patterns of drug use in the neonates were determined.

### Exclusion criteria

Reviews, editorials, comments, clinical trials, book chapters, conference abstracts, and other studies relating to other age categories including children (2–11 years old), adolescents (12–17 years old), and adults (≥18 years old) were excluded. Studies related to a higher age limit (>23 months) and those involving outpatient children or children attending emergency departments were excluded due to the high possibility of variation with regard to medical conditions and pharmacotherapy prescribed. Studies evaluating adverse drug reactions, costs or health-care resource utilization were excluded.

### Selection of the studies

After the removal of duplicates, the identified publications were manually screened on the basis of titles and abstracts. Observational studies identified as containing potentially relevant information were subsequently reviewed as full-text articles. To further enhance the search, the reference lists of the selected publications were checked for other relevant publications that might meet the study eligibility criteria. Unpublished or gray literature was excluded.

The selected full-text articles were evaluated for relevancy and quality of study design. In order to assess the risk of potential bias, the quality of selected studies was assessed using 14 criteria of the Quality Assessment tool for Observational Cohort and Cross-sectional Studies proposed by National Institutes of Health (United States), 2016. For each item on the tool, the reviewer could select “yes,” “no,” or “cannot determine/not reported/not applicable.” The potential risk of bias was considered if “no” or “cannot determine/not reported/not applicable” were selected for the items by the reviewer. Reviewers then rated the quality of the study as “good,” “fair,” or “poor.” Studies that were categorized as “good” have the least risk of bias' “fair” studies have an intermediate risk of bias. Studies with a “poor” rating had a significant risk of bias and were thus excluded from further evaluation. Meta-analysis was not performed as the studies retrieved were heterogeneous and were all observational study designs.

### Data extraction

A data extraction sheet was designed to guide the evaluation process of the selected articles and to capture information on the variables of interest. The eligibility assessment was performed independently by two reviewers. The first reviewer, indicated as RR, screened titles and abstracts, and checked the eligibility of abstracts and full text after initial screening for data extraction. A second reviewer, indicated as LCM, checked the information tabulated in the data synthesis matrix with the full text articles included in the review. The eligibility of the studies included was also assessed by the second reviewer and there were no disagreements in eligibility assessment between the two reviewers. Details of study design, study objectives, study population and sample size, study setting, methods on quantifying drug consumption, and the main findings were extracted from the articles. If available, information on the interventions or recommendations to improve reporting or rational drug use was also retrieved.

## Results

### Search results

The initial search of the four databases resulted in the retrieval of 4389 articles. However, due to the duplication of articles retrieved from CINAHL, Embase, and PubMed from those found through Medline, almost all articles from these search engines were excluded from the study. As shown in Figure 1, after the screening of the titles and abstract, 52 articles remained for further evaluation of the full text. Out of the 52, the following were not considered for in-depth analysis of drug utilization: studies that involved neonates or infants with particular conditions (*n* = 1); studies evaluating off-label/unlicensed drug use (*n* = 3); studies analyzing drug use of a specific drug(s) (*n* = 8) or a category of drug (excluding antimicrobials) (*n* = 13); studies analyzing drug use in comparison with the drug expenditure (*n* = 2); and studies on drug utilization in the general pediatric population (*n* = 3).

The remaining 20 articles were included in the review (Medline, *n* = 14, and Embase, *n* = 2, and from manual searching through other databases, World of Science, *n* = 3, and Google Scholar, *n* = 1). Out of the 20 selected articles, 12 studies evaluated all drug categories in neonates, whilst the others evaluated antimicrobials used in neonates. Studies evaluating antimicrobials were included as these agents were the most commonly prescribed in neonates. Three articles (Kumar et al., 2008; Neubert et al., 2010; Lass et al., 2011) partly evaluating unlicensed drug/off-label use were included as these studies also reported overall drug consumption in neonates. For drug utilization study on antibiotics, four studies involved other pediatric age groups (Grohskopf et al., 2005; Zingg et al., 2011; Porta et al., 2012; Salehifar et al., 2014). No articles related to drug utilization in neonates were found from cross-reference of the bibliographies of the full-text articles selected.

### Quality of studies

A quality assessment of the selected studies indicated that 15% (*n* = 3) were poor quality, 45.0% (*n* = 9) were fair, and 40.0% (*n* = 8) were of good quality. The National Institutes of Health Quality Assessment guidelines recommend excluding poor-quality articles from further analysis. However, in this review, these articles were included as the total number of retrieved articles was low (*n* = 20).

### Characteristics of the drug utilization studies

The general characteristics of selected studies included are summarized in Table 2. Of the 20 selected articles, 35% (*n* = 7) were from Europe [Estonia (*n* = 1), Germany (*n* = 1), Poland (*n* = 2), Switzerland (*n* = 1), the Netherlands (*n* = 1), and Multinational (*n* = 1) (Greece, United Kingdom, Italy)] 30% were from the United States (*n* = 6), 25% were from South Asia (India, *n* = 5) and single studies reported on South America (Brazil) and the Middle East (Iran). No publications related to drug consumption in neonates from other regions, including Southeast Asia, were identified. The distribution of studies was uneven over a 12-year period (2005–2016), with only six studies published from 2005 to 2008. 70% (*n* = 14) of the studies were published from 2010 onwards. The observation periods ranged from 1996 to 2014, with the study duration ranging from 2 days to 9.3 years.

**Table 2:** **Characteristics of the included studies**.

**Study Type/Year Published**	**Data source**	**Country/Setting**	**Timeframe (Month/Year)**	**Study period**	**No. of subjects, n**	**References**
**ALL DRUGS**						
Prospective observational (2016)	Medical Record/Prescription	India (NICU)	Apr–Sept 2014	6 months	528 neonates	Suryawanshi et al., 2016
Prospective observational (2015)	Medical record	Brazil (NICU)	Jan–June 2012	6 months	187 neonates	Goncalves et al., 2015
Prospective observational (2015)	Medical record	India (NICU)	Mar 2013–Feb 2014	1 year	650 neonates	Patel Brijal et al., 2015
Retrospective review (2014)	Administrative database	United States (350 NICUs)	Jan 2005–Dec 2010	6 years	450,386 infants	Hsieh et al., 2014
Prospective cohort (2011)	Prescription	Estonia (NICU, neonatal wards) (2 Hospitals)	Feb–Aug 2008 Feb–Aug 2009	1 year	490 neonates	Lass et al., 2011
Prospective, cohort (2010)	Medical Record/Prescription	Germany (NICU)	Dec 2004–Oct 2005	11 months	183 neonates	Neubert et al., 2010
Retrospective (2008)	Pharmacy dispensing database	United States (NICU)	Sept 2000–Aug 2003	3 years	2304 neonates	Kumar et al., 2008
Prospective observational (2007)	Medical Record/Prescription	India (NICU)	Mar–Aug 2005	6 months	176 neonates	Chatterjee et al., 2007
Retrospective cohort analysis (2006)	Neonatal database	United States (NICU, PCN)	Jan 1997–Dec 1998 Jan 2001 Jun 2004	5.5 years	5023 neonates	Du et al., 2006
Retrospective cohort (2006)	National database	United States (NICU) (220 hospitals)	Jan 1996–Apr 2005	9.3 years	253651 reports	Clark et al., 2006
Retrospective (2006)	Neonatal database	United States (NICU & PCN)	Jan 1997–June 2004	7.5 years	6839 neonates	Warrier et al., 2006
No information (2014)	Medical records	India (NICU)	Jan–June 2013	6 months	100 neonates	(Sharanappa et al., 2015)
**ANTIBIOTICS**						
Prospective (2015)	Prescription	India (NICU)	Feb–Apr 2013	3 months	250 prescriptions	Subash and Shanmugapriyan, 2015
Prospective cohort (2011)	Patient chart	Switzerland (NICU, PICU)	Apr 2001–Dec 2008	7.7 years	1096 neonates	Zingg et al., 2011
Point prevalence survey (2012)	Survey	UK, Greece& Italy (Neonatal & Paediatric ward) (4 hospitals)	Feb–May 2009	14 days	1217 prescription (269 neonates)	Porta et al., 2012
Point prevalence surveys (2005)	Survey	United States (NICU, PICU) (31 hospitals)	Aug 1999–Feb 2000	2 days	2647 patients (1580 neonates)	Grohskopf et al., 2005
Retrospective analysis (2015)	Pharmacy Purchase Data	Poland (SNCU)	Jan 2011–Dec 2012	2 years	418 neonates	Nitsch-Osuch et al., 2015
Retrospective (2013)	Pharmacy Purchase Data	Poland (SNCU)	Jan–Dec 2011	1 year	801 records	Nitsch-Osuch et al., 2013
Retrospective (2010)	Pharmacy Purchase Data	Netherland (10 NICUs)	Jan–Dec 2005	1 year	4326 patients	Liem et al., 2010b
Cross sectional (2014)	HIS database	Iran (Neonatal, NICU, PICU, Paediatrics, Paediatric surgery)	Sept 2010–Sept 2011	1 year	4619 records (765 neonates)	Salehifar et al., 2014

*NICU, Neonatal Intensive Care Unit; PCN, Progressive Care and Observation Nurseries; PICU, Paediatric Intensive Care Unit; SCNU, Special Neonatal Care Unit*.

Overall, eight studies were prospective (3 cohort studies), 9 were cross-sectional or retrospective (2 cohort studies), and two were point prevalence surveys. We failed to determine the kind of study design of one article, and we deemed it as poor quality. The data sources were mainly medical records or patient charts and/or prescriptions (*n* = 9), followed by administrative databases that were part of periodical health care monitoring systems (*n* = 5) and pharmacy purchase databases (*n* = 3). Other studies utilized standardized questionnaires (*n* = 1) and pharmacy dispensing databases (*n* = 1). Studies using data derived from a database were mainly retrospective in nature, with a longer period of study coverage, ranging from 1 year to 9.3 years. On the other hand, studies conducted using data from patient medical records and drug prescriptions that were conducted prospectively had shorter study coverage, ranging from 3 months to 1 year. Others were point prevalence surveys using questionnaires and prescriptions, which were carried out in 2 and 14 days respectively, and a study using patients' charts, with a coverage of 7.7 years.

### Objectives of selected studies

A majority of the selected studies (70%) aimed to describe drug consumption patterns, with 15% of the studies evaluating changes in drug utilization patterns in neonates. Other objectives include evaluation of licensing status or off-label use of drugs (15%), determination of future research areas (10%) and evaluation of the impact of intervention (10%). The full list of objectives is shown in Table 3. Some of the studies had more than one objective.

**Table 3:** **Study objectives of the selected studies**.

**Study objectives**	**Studies**		
	n^*^	**%**	**References**
Drug utilization review	14	70	Grohskopf et al., 2005; Clark et al., 2006; Warrier et al., 2006; Chatterjee et al., 2007; Kumar et al., 2008; Liem et al., 2010b; Neubert et al., 2010; Lass et al., 2011; Nitsch-Osuch et al., 2013; Salehifar et al., 2014; Goncalves et al., 2015; Sharanappa et al., 2015; Subash and Shanmugapriyan, 2015; Suryawanshi et al., 2016
Changes in drug utilization pattern	3	15	Clark et al., 2006; Du et al., 2006; Hsieh et al., 2014
Licensing status/ off label use	3	15	Kumar et al., 2008; Neubert et al., 2010; Lass et al., 2011
Determination of critical area for future research	2	10	Warrier et al., 2006; Neubert et al., 2010
Study impact of intervention	2	10	Zingg et al., 2011; Nitsch-Osuch et al., 2015
Drug utilization review and ADRs	1	5	Patel Brijal et al., 2015
Association of perinatal care, clinical care and drug use	1	5	Goncalves et al., 2015
Development of methodology	1	5	Porta et al., 2012
Evaluation of different methods in reporting	1	5	Clark et al., 2006

* *Some studies had more than one objectives, although 20 studies were selected, information was extracted on objectives was 28 and percentages do not add up to 100%*.

### Methods used in drug utilization studies

As shown in Table 4, a variety of methods were used in the selected studies to present data on drug utilization. Some studies utilized more than one method. More than half of the selected studies, mainly describing drug consumption for all category of drugs, reported the prevalence of drug used in terms of the number of patients or prescriptions, which stated as either count, courses, exposure, or exposure rate. Interestingly, three studies from India reported drug consumption in neonates using WHO core prescribing indicators (Chatterjee et al., 2007; Patel Brijal et al., 2015; Suryawanshi et al., 2016).

**Table 4:** **Methods, samples, percentage and type of drugs used in the included studies**.

**No**.	**Data Source**	**Methods**	**Results**	**References**
**ALL DRUGS**				
1.	Medical record	Exposure rate by gestational age (number of prescription, percentage of prescription) ATC system	Total neonates: 187 Multivitamin, *n =* 100 (53.5%), gentamicin, *n =* 78 (41.7%), ampicillin, *n =* 66 (35.3%), fentanyl, *n =* 66 (35.3%), midazolam, *n =* 60 (32.1%)	Goncalves et al., 2015
2.	Medical records	Number of neonates Percentage of neonates	Number of neonates: 100 Ceftriaxone, *n =* 75 (75.0%), amikacin, *n =* 75 (75.0%), phenobarbitone, *n =* 39 (39.0%), phytomenadione, *n =* 19 (19.0%), aminophylline, *n =* 11, (11.0%)	Sharanappa et al., 2015
3.	Medical records	Number of neonates Percentage of neonates Drug category WHO core prescribing indicator	Number of neonates: 650 Phytomenadione, *n =* 471 (72.5%), amikacin, *n =* 251 (38.6%), cefotaxime, *n =* 222 (34.2%), ampicillin, *n =* 199 (30.6%), gentamicin, *n =* 197 (30.3%), metronidazole, *n =* 102 (15.7%), adrenalin, *n =* 77 (11.9%), dopamine, *n =* 42 (6.5%), phenobarbitone, *n =* 61 (9.4%)	Patel Brijal et al., 2015
4.	Prescription	Number of prescriptions by gestational age Prescription rates by gestational age (no. of prescription/100 admissions) ATC	Number of neonates: 490 Gentamicin, *n =* 200 (40.8%), ampicillin, *n =* 92 (18.8%), fentanyl, *n =* 87 (17.8%), frusemide, *n =* 81 (16.5%), heparin, *n =* 26 (17.5%)	Lass et al., 2011
5.	Medical Record/Prescription	Number of prescriptions by gestational age Exposure rate (percentage) ATC	Number of neonates: 183 Phytomenadione, *n =* 163 (89.1%), piperacillin, *n =* 147 (80.3%), tobramycin, *n =* 146 (79.8%), theophylline, *n =* 95 (51.9%), midazolam, *n =* 89 (48.6%), diazepam, *n =* 44 (24.0%), caffeine, *n =* 38 (20.8%), surfactant, *n =* 35 (19.1%)	Neubert et al., 2010
6.	Medical Record/Prescription	ATC WHO core prescribing indicators	Total prescription: 849 Antimicrobials, *n =* 256 (30.2%) Vitamins, *n =* 176 (20.7%)	Chatterjee et al., 2007
7.	Medical Record/Prescription	Number of prescription by gestational age Percentage of prescription ATC & active substance by gestational age WHO core prescribing indicators	Total prescription: 1658 Antibiotics, *n =* 1123 (67.7%) Respiratory system, *n =* 157 (9.5%) Nervous system, *n =* 149 (9.0%) Cardiovascular system, *n =* 115 (6.0%) Alimentary tract system, *n =* 52 (3.1%)	Suryawanshi et al., 2016
8.	Administrative database	Counts & Proportions by 3 methods: Exposure (number of unique medication names reported for each patient) Total medication courses (number of times a unique medication name reported) Days of use (total number of days each medication was administered)	Unit reported as exposure per 1000 infants: Ampicillin (681), gentamicin (676), caffeine citrate (156), beractant (91), vancomycin (91), beractant (82), frusemide (81), fentanyl (70), dopamine (62), midazolam (61) Increased use: azithromycin, sildenafil, milrinone Decreased use: theophylline, doxapram, metoclopramide	Hsieh et al., 2014
9.	Pharmacy dispensing database	Number of neonates on medication Number of doses dispensed Drug category	Total neonates: 2304 Ampicillin, *n =* 1691 (73.4%), gentamicin, *n =* 1667 (72.4%), heparin, *n* = 1075 (46.7%), surfactant, *n =* 540 (23.4%), fentanyl, *n =* 446 (19.4%), caffeine, *n =* 374 (16.2%), indomethacin, *n =* 311 (13.5%), frusemide, *n =* 268 (11.6%), erythropoietin, *n =* 250 (10.9%), dopamine, *n =* 213 (9.2%)	Kumar et al., 2008
10.	Neonatal database	Mean number of drug per infant Drug category	Increased use: Antibiotics, central nervous system drugs, endocrine agents, cardiovascular, gastrointestinal drugs Decreased utilization: ophthalmic drugs No changes: nutritional, biological, renal, pulmonary drugs	Du et al., 2006
11.	National database	Counts: Frequency (number of times a unique medication name reported) Courses (number of times a unique medication was reported for a single patient with a specific start date) Exposure (number of unique medication names reported for each patient) Exposure rate (exposure/total study cohort x1000)	Unit reported as exposure rate: Ampicillin (693), gentamicin (575), ferrous sulfate (269), multivitamin (228), cefotaxime (183), caffeine citrate (130), frusemide (84), vancomycin (99), surfactant (118), metoclopramide (82)	Clark et al., 2006
12.	Neonatal database	Exposure rate by gestational age (percentage of infant exposed) Mean number of drug per infant Drug category	Unit reported as exposure rates: Ampicillin (94.2%), cefotaxime (92.4%), beractant (18.2%), dopamine (10.1%), iron (9.5%), vancomycin (8.8%)	Warrier et al., 2006
**ANTIBIOTICS**				
13.	Prescription	Number of prescription Percentage of prescription	Total prescriptions: 250 Ampicillin, *n =* 55 (21.7%), gentamicin, *n =* 55 (21.7%), amikacin, *n =* 50 (20.1%), piperacillin/tazobactam, *n =* 39 (15.8%), cefotaxime, *n =* 28 (11.4%)	Subash and Shanmugapriyan, 2015
14.	Patient chart	Days Of Therapy (DOT)/1000 patient days	Days of therapy were 360 per 1000 patient-days	Zingg et al., 2011
15.	Prescription	Number of prescriptions or patients PDD/100 bed days DDD/100 bed days -DU90% Doses (mg/kg/day) ATC	^*^Total prescriptions: 263 Penicillin (benzylpenicillin, flucloxacillin), *n =* 101 (38.4%) Aminoglycosides (gentamicin, amikacin), *n =* 90 (34.2%) Other antibacterials (vancomycin, metronidazole), *n =* 49 (18.6%)	Porta et al., 2012
16.	HIS database	DDD/100 inhabitant per day (DID)	^*^Unit reported as DID: Ampicillin (64.3), amikacin (17.2), cefotaxime (6.0), cefepime (4.8), meropenem (4.9), vancomycin (8.1)	Salehifar et al., 2014
17.	Pharmacy purchase data	DDD/100 patient-days DDD/100 admissions DU90% & DU 100%	^*^Unit reported as DDD/100 admissions: Ampicillin (367.2), amoxicillin/clavulanic acid (146.6), amikacin (24.7), gentamicin (25.1)	Nitsch-Osuch et al., 2015
18.	Pharmacy purchase data	DDD/100 admission ATC DU 90%	Antibiotic consumption ranged from 130 to 360 DDD/100 admissions	Liem et al., 2010b
19.	Pharmacy purchase data	DDD/100 patient days DDD/100 admissions ATC DU90% & DU100%	Unit reported as DDD/100 admissions: Ampicillin (136.9), amoxicillin/ clavulanic acid (143.3), amikacin (22.4), gentamicin (13.2)	Nitsch-Osuch et al., 2013
20.	Questionnaire	Number of patients on medication Frequency of drug use	^*^Total neonates: 1580 Gentamicin, *n =* 354 (22.3%), ampicillin, *n =* 324 (20.4%), vancomycin, *n =* 174 (10.9%), cefotaxime, *n =* 105 (6.6%)	Grohskopf et al., 2005

* *Only data on neonates reported*.

Table 4 shows that the ATC/DDD system was only used in three studies evaluating antibiotics (Liem et al., 2010b; Porta et al., 2012; Nitsch-Osuch et al., 2013). Two studies used the DDD system without ATC classification, whereas one study reported antibiotics consumption using Days of Therapy (Zingg et al., 2011; Salehifar et al., 2014; Nitsch-Osuch et al., 2015). Only one study reported antibiotics consumption using the PDD system (Porta et al., 2012). In addition, DU90 or DU100% was used as one of the reporting methods in four studies evaluating antibiotic utilization (Liem et al., 2010b; Porta et al., 2012; Nitsch-Osuch et al., 2013, 2015).

For drug classification, only five studies evaluating all drugs utilized ATC for drug classification, whilst others used non-standardized classification of pharmacological groups (Chatterjee et al., 2007; Neubert et al., 2010; Lass et al., 2011; Goncalves et al., 2015; Suryawanshi et al., 2016). A few studies reported specific drugs commonly prescribed for neonates instead of using a drug classification system for reporting (Grohskopf et al., 2005; Clark et al., 2006; Warrier et al., 2006; Hsieh et al., 2014; Sharanappa et al., 2015; Subash and Shanmugapriyan, 2015).

### Prescribing pattern

The most commonly reported drugs across all selected studies were anti-infectives for systemic use, followed by drugs for the cardiovascular system, the nervous system and the respiratory system. The range of antibiotics reported in studies evaluating antibiotics were in agreement with the findings from studies evaluating all drug consumption in neonates. The most common pharmacological groups reported were Penicillin and aminoglycosides, followed by other beta lactam antibiotics. Ampicillin and gentamicin were the most frequently used agents in neonates and were reported in the majority of the studies. The detailed list of common drugs reported in the selected studies is shown in Table 4. Five studies documented drug consumption and its comparisons across neonatal gestational age groups (Warrier et al., 2006; Neubert et al., 2010; Lass et al., 2011; Goncalves et al., 2015; Suryawanshi et al., 2016).

## Discussion

The number of drug utilization studies in neonates is limited, with 12 studies investigating the overall usage of drugs (all categories) and eight studies focused solely on antibiotic usage. The majority of the studies were carried out in Europe and the United States, dwarfing other studies from other regions. The possible reason for the high number of drug utilization studies in European countries could be due to initiatives taken by the countries in establishing research working groups on drug utilization, in effort to assess the benefit risk of medicines. The Task Force in Europe for Drug Development for the Young (TEDDY) for example is one of the research working groups that actively conduct drug utilization research to promote safe and effective drugs in pediatrics (Sabate et al., 2014). No information on drug utilization research working groups for other countries was identified.

In order to perform a robust DUR, reliable information such as cumulative data from administrative databases (local, regional, or national) and pharmacy purchase data or pharmacy dispensing database, patient medical records, or charts and drug prescriptions is needed. Similar to the review conducted by Clavenna and Bonati, the use of different data sources contributed to the heterogeneity (study designs, sample size, and data collected) found in this review (Clavenna and Bonati, 2009). Cohort studies have been used to study drug utilization patterns over time, as revealed in this review (five cohort studies, Verhamme and Sturkenboom, 2011). Due to variation in study types (design and methods), data collected and methods used for reporting drug consumption, comparative evaluation of studies was difficult.

DUR with the use of a standardized methodology (WHO ATC/DDD) allows a researcher to compare drug use in different countries and other levels of healthcare on an equal basis. It must be noted that the ATC classification system is now used as an international standard to classify drugs. DDD denotes the average maintenance dose of the drug when used on its major indication in adults (World Health Organization, 2003). DDD serves as a technical unit of measurement in DUR. The issue in assessing neonatal drug utilization patterns is the unavailability of a common methodology for quantifying drug consumptions as the DDDs proposed were meant for adults (Lass et al., 2011). From the present review, only studies evaluating antibiotics utilized ATC/DDD methods. Although the DDD methodology is widely used in adults, its applicability in neonates is yet to be studied or validated. This is because dosing in children is calculated based on their body weight and for the purpose of DDD calculation, an average of body weight for the pediatric population needs to be assumed. However, pediatric DDDs still remain questionable because dose recommendations in children, especially neonates, vary according to their age and body weight (Monnet, 2007; Liem et al., 2010a).

Other methods used for the evaluation of drug utilization were PDD, Days of Therapy (DOT) and DU 90%. PDD denotes an average dose prescribed according to a representative sample of prescriptions. PDD emphasizes the amount of specific drugs consumed (World Health Organization, 2003; Porta et al., 2012). Technically, DOT is the number of days that at least one dose of the drug is taken or should have been taken (Polk et al., 2007). In other words, one DOT represents the administration of a single agent on a given day, regardless of the number of doses administered or dosage strength. Previous DUR suggested that DOT methodology is useful to reflect drug usage in the pediatric population as its calculation is not affected by the difference between DDD and PDD or changes in WHO-assigned DDD. Most importantly, the DOT methodology is independent of age- or weight-related differences in dosage (Polk et al., 2007; Liem et al., 2010b). However, the use of PDD and DOT were not popular as only a single study evaluated antibiotic consumption using these methods (Zingg et al., 2011; Porta et al., 2012). The DU90% was used in four of the included studies, and it is an essential tool to assess the quality of drug prescription. The DU90% represents the number of drugs accounting for 90% of drug use (Bergman et al., 1998).

Knowing the limitations of applying DDD to pediatrics, especially neonates, three studies from India utilized WHO core prescribing indicators for reporting drug consumption. The WHO core prescribing indicators is a set of drug-prescribing indicators that measure: the degree of polypharmacy; the tendency to prescribe drugs by generic name; the overall level of use of antibiotics and injections; and the degree to which the prescribing practice conforms to the essential drug list, formulary or standard treatment guideline (World Health Organization, 1993). Although these indicators were developed to evaluate the appropriateness of drug use in outpatient health facilities, in an effort to promote rational drug use, they have also been used in studies evaluating drug use for inpatient children (Woldu et al., 2013; Ambaw and Gabriel, 2015). Nevertheless, as drug use patterns are more complex in inpatient settings, or in specialty outpatient clinics in referral hospitals, these indicators appear to be less useful when applied to NICU or other neonatal wards (World Health Organization, 1993).

The majority of the selected studies utilized a volume unit, such as the number of prescriptions, drugs or patients, to compute the consumption of drugs in general neonates. Although these volume units can be used to make a national comparison of drug consumption, none of them are applicable for cross-national comparisons (World Health Organization, 2003). Several attempts have been made by researchers to propose a valid and reliable methodology for drug utilization measurement in neonates. From a pilot study in four European children's hospitals located in the UK, Greece and Italy, a 3-step algorithm for reporting antimicrobial drug utilization in pediatric patients has been produced. However, the algorithm need to be validated in larger populations (Porta et al., 2012). Another piece of research demonstrated that a set of neonatal DDDs for 10 commonly used antibiotics based on an assumed neonatal weight of 2 kg could be a useful study methodology (Liem et al., 2010a).

The International Conference on Harmonisation Guidelines on Clinical Investigation of Medicinal Products in the Paediatric Population is well aware of the elevated level of clinical research and has recommended that drug effects within different age categories in children are studied (International Conference on Harmonisation of Technical Requirements for Registration of Pharmaceuticals for Human Use, 2014). Indeed, in neonates, pattern of drugs differs based on gestational age and this has been documented in studies included in this review (Warrier et al., 2006; Neubert et al., 2010; Lass et al., 2011; Goncalves et al., 2015; Suryawanshi et al., 2016). As antibiotics are crucial to ensure survival after a serious infection, antimicrobial agents for systemic use (ampicillin and gentamicin) are the most frequently used therapeutic agents in this group of patients.

Like other systematic review, this review has its own limitations. For example, non-English language articles on neonatal drug utilization neonates could have been missed as they were excluded. Secondly, although a complete search was conducted, only studies evaluating all drug categories and the most common therapeutic drug class were analyzed, while some drug utilization studies on other classes were carried out in neonates. We cannot perform a meta-analysis due to the vast heterogeneity of the data reported, notwithstanding different study design and measuring parameters. Nevertheless, in order to enhance the quality of the review, gray literature was not included and only the best-quality studies were evaluated for review.

## Conclusions

The present review revealed that neonates are exposed to a high number of drugs. Various methods were used to report drug consumption in this age group. The quality assessment shows that 45 and 40% of the included studies are classified as fair and good quality, respectively. An equal percentage was seen in terms of the study design used, namely prospective, cross-sectional and retrospective studies. Up to 70% of the selected studies aimed to describe drug consumption patterns of neonates. The best measure of drug consumption to adequately quantify prevalence of drug use in neonates remains to be identified, and additional research in this field is warranted.

## Data availability statement

All relevant data are within the paper.

## Author contributions

RR conducted data collection. RR, AD, LM, NA, AA, and MM were involved in data analyses and interpretation of the results. All authors participated in research design, contributed to the writing of the manuscript and approved the final manuscript.

### Conflict of interest statement

The authors declare that the research was conducted in the absence of any commercial or financial relationships that could be construed as a potential conflict of interest.
